# Genetic engineering of *Yarrowia lipolytica* for enhanced production of *trans*-10, *cis*-12 conjugated linoleic acid

**DOI:** 10.1186/1475-2859-12-70

**Published:** 2013-07-16

**Authors:** Baixi Zhang, Haiqin Chen, Min Li, Zhennan Gu, Yuanda Song, Colin Ratledge, Yong Q Chen, Hao Zhang, Wei Chen

**Affiliations:** 1 State Key Laboratory of Food Science and Technology, Jiangnan University, 1800 Lihu Ave, Wuxi, Jiangsu 214122, P.R. China; 2 School of Food Science and Technology, Jiangnan University, Wuxi 214122, P.R. China; 3 Department of Biological Sciences, University of Hull, Hull HU6 7RX, UK

**Keywords:** Conjugated linoleic acid, Fermentation, Multi-copy integration, *opai*-*d12* co-expression, Promoter hp16d, Soybean oil, *Yarrowia lipolytica*

## Abstract

**Background:**

Conjugated linoleic acid (CLA) has been extensively studied for decades because of its health benefits including cancer prevention, anti-atherogenic and anti-obesity effects, and modulation of the immune system. We previously described the production of *trans*-10, *cis*-12 CLA in *Yarrowia lipolytica* by expressing the gene coding for linoleic acid isomerase from *Propionibacterium acnes* (*pai*). However the stable strain produced CLA at about 0.08% of dry cell weight (DCW), a level of production which was not high enough for practical applications. The goal of the present study was to enhance production of CLA by genetic engineering of *Y*. *lipolytica* strains.

**Results:**

We have now co-expressed the delta 12-desaturase gene (*FADS12*, *d12*) from *Mortierella alpina* together with the codon-optimized linoleic acid isomerase (*opai*) gene in *Y*. *lipolytica*, expressed under the control of promoter hp16d modified by fusing 12 copies of UAS1B to the original promoter hp4d. A multi-copy integration plasmid was used to further enhance the expression of both genes. Using glucose as the sole carbon source, the genetically-modified *Y*. *lipolytica* produced *trans*-10, *cis*-12-CLA at a level of up to 10% of total fatty acids and 0.4% of DCW. Furthermore, when the recombinant yeast was grown with soybean oil, *trans*-10, *cis*-12-CLA now accumulated at a level of up to 44% of total fatty acids, which represented 30% of DCW after 38.5 h of cultivation. In addition, *trans*-10, *cis*-12-CLA was also detected in the growth medium up to 0.9 g/l.

**Conclusions:**

We have successfully produced *trans*-10, *cis*-12-CLA with a titre of 4 g/l of culture (3.1 g/l in cells and 0.9 g/l in culture medium). Our results demonstrate the potential use of *Y*. *lipolytica* as a promising microbial cell factory for *trans*-10, *cis*-12-CLA production.

## Background

Conjugated linoleic acids (CLA) are geometric and positional dienoic isomers of linoleic acid (18:2, n-9). Over the last three decades, CLA has been extensively studied because of its health benefits including cancer prevention, anti-atherogenic and anti-obesity effects, and immune system modulation [1]. The multiple beneficial physiological effects of CLA can be attributed to the two most studied isomers, with *cis*-9, *trans*-11 CLA playing the most important role in the anticancer effects, and *trans*-10, *cis*-12 CLA having a predominant effect on weight management and prevention of atherogenesis [2,3]. For the purpose of research and applications in medicinal and nutritional fields, each CLA isomer needs to be available at high purity and in large quantity. However, the chemically-produced CLA is a mixture of four (8,10-, 9,11-, 10,12-, and 11,13-18:2) *cis*/*trans* positional isomers [4]. As a result, biological processes to produce single CLA isomers have been investigated [5-10]. To date, the highest yield of *cis*-9, *trans*-11 CLA (10.5 mg/ml) was obtained in *Delacroixia coronata* using *trans*-vaccenic acid methyl ester as substrate [6]. By contrast, the bioprocess yield of the other isomer, *trans*-10, *cis*-12 CLA was very low, with a mere 0.2 mg/ml in recombinant *Escherichia coli* using exogenous linoleic acid (LA) as substrate [9].

In our previous study [10], we constructed a *de novo trans*-10, *cis*-12 CLA biosynthesis system by transforming the oleaginous yeast, *Yarrowia lipolytica*, with the recombinant linoleate isomerase gene (*pai*) from *Propionibacterium acnes*. Codon usage optimization of *pai* and multi-copy integration significantly improved the expression of the linoleate isomerase gene in *Y*. *lipolytica*. The amount of *trans*-10, *cis*-12 CLA reached 5.9% of total fatty acids, and 0.23% of DCW [10]. However, the yeast strain with the highest integrated copy number (24 copies) was unstable and the yield of CLA gradually decreased to 0.08% of DCW. This yield therefore was not high enough for further development. In an attempt to further increase *trans*-10, *cis*-12 CLA production, we genetically modified *Y*. *lipolytica* to improve the expression of linoleate isomerase gene, and modified fermentation conditions to increase the concentration of the isomerase substrate, LA. In this manner, the yield of *trans*-10, *cis*-12 CLA was improved significantly.

## Results

### Construction of recombinant yeast strains

Polh-pINA1312-*opai*, the single-copy integration strain with the codon-optimized *opai* gene described in our previous study [10], was used as a control and as a model strain to develop CLA high-producing recombinant strains. To increase LA content in *Y*. *lipolytica*, the delta12-desaturase gene (*d12*) was cloned from *M*. *alpina* to construct *opai* -*d12* co-expression cassettes. Delta12-desaturase converted oleic acid (18:1) to LA (18:2). Polh-pINA1312-*opai*-*d12* was obtained by transforming Polh with *opai*-*d12* co-expression cassettes. To enhance *opai* expression, the promoter, hp4d, was replaced by a modified promoter, hp16d, located upstream of *opai*, resulting in the recombinant strains Polh-pINA1312-sp*opai* and Polh-pINA1312-sp*opai*-*d12*. Furthermore, the sp*opai* -*d12* co-expression cassettes were also placed in a multi-copy integration plasmid, pINA1292. The new multi-copy integration cassette was transformed to obtain the strain Polh-pINA1292-sp*opai*-*d12*. Eight to twelve transformants in each series were picked randomly and confirmed to be all positive by PCR analysis.

### *opai* / *d12* copy numbers in Polh-1292-sp*opai*-*d12* transformants

**Figure 1:**
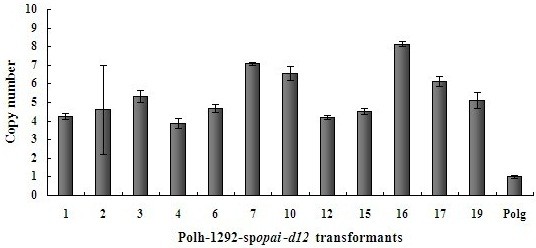
**Relative copy numbers of integrated expression cassettes in *Y. lipolytica *multi**-**copy transformants. **Real-time PCR was used to estimate the copy numbers of the integrated expression cassettes among 12 Polh-1292-sp*opai*-*d12* transformants. *Y*. *lipolytica* Polg was used as a control organism with a single copy of both the *ura3* and *suc2* target sequences. As *ura3*, *opai* and *d12* coexisted in expression cassettes, the copy numbers of three genes were considered to be equal. Error bars represent standard deviations from biological triplicates.

Twelve selected transformants with the multi-copy integration cassette derived from plasmid pINA1292-sp*opai*-*d12* were investigated. The copy numbers of the co-expression cassettes were estimated using the data obtained by real-time PCR analysis. *Y*. *lipolytica* Polg was used as a control organism with a single copy of both *ura3* and *suc2* target sequences. As *ura3*, *opai* and *d12* coexisted in expression cassettes, the copy numbers of three genes were considered to be equal. Distribution of o*pai* / *d12* copy numbers analyzed in twelve isolated transformants is shown in Figure 1. For all the clones tested, copy numbers fell in a narrow range of four to eight copies, with an average of five to six copies/cell. The strain with the highest copy number was Polh-pINA1292-sp*opai*-*d12*-16 (8 copies).

### Effect of genetic modifications on *opai* transcript and protein levels

**Figure 2:**
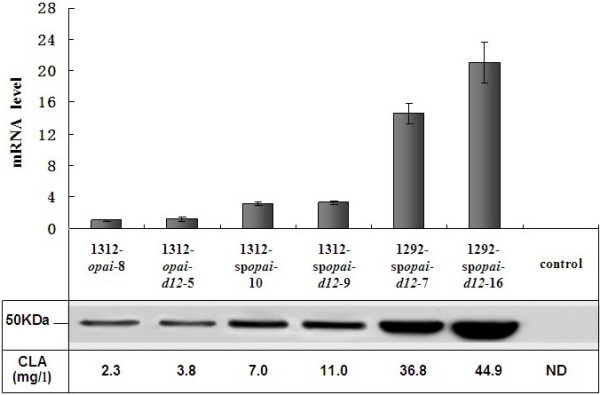
**Transcript and protein levels of recombinant *PAI *and CLA yield in different *Y. lipolytica *strains. **For comparative analysis of opai transcription and protein expression levels, five representative transformants with an average CLA yield in each series were selected, along with the best-performing transformant, Polh-pINA1292-spopai-d12-16. The *Y*. *lipolytica* strains were harvested after 72 h cultivation in YPD medium. Transcript levels were determined by real-time PCR and protein levels were analyzed by western blot analysis using specific polyclonal antibodies. The control used was the strain transformed with the empty vector pINA1312. The standard deviations were < 5% of the values shown.

Lipids and biomass of all transformants grown in YPD medium were determined. For comparative analysis of *opai* transcript and protein levels, five representative transformants with an average CLA yield in each series were selected, along with the best-performing transformant, Polh-pINA1292-sp*opai*-*d12*-16. Polh-pINA1312-*opai*-8 [10] was used as the control strain. The *opai* transcript levels in Polh-pINA1312- *opai*-*d12*-5 were similar to those in the control strain (Figure 2). This suggested that *d12* overexpression driven by promoter hp4d did not affect *opai* transcription. In contrast, the transcription levels of *opai* in Polh-pINA1312-sp*opai*-10 and Polh-pINA1312-sp*opai*-*d12*-9 were 3-fold higher than in the control strain (Figure 2), which demonstrated that the modified promoter hp16d was stronger in eliciting *opai* transcription than hp4d. In addition, there was a 14.6-fold increase in *opai* transcript levels in the multi-copy integrated strain Polh-pINA1292-sp*opai*-*d12*-7 (Figure 2), suggesting that multi-copy integration could also increase *opai* levels due to a gene dosage effect. As expected, the best-performing transformant, Polh-pINA1292-sp*opai*-*d12*-16, had the highest *opai* mRNA level, corresponding to a 21-fold increase compared with the control strain (Figure 2). The *opai* protein levels in the six strains showed a good correlation with the transcript levels (Figure 2).

### Effect of genetic modifications on CLA production

To determine whether the above-described genetic modifications had a positive effect on CLA production, the recombinant *Y*. *lipolytica* strains were cultured in YPD for 72 h at 28°C. Lipids were then extracted and analyzed by GC. CLA yields in different *Y*. *lipolytica* strains are shown in Figure 2. Compared to the control strain Polh-pINA1312-*opai*-8, expression of the heterologous *d12* and promoter modification increased CLA production by 1.7- and 3-fold, respectively. Furthermore, the combined effect of *d12* expression and modified promoter resulted in a 4.8-fold increase in CLA yield compared to the control. The multi-copy integration of o*pai* also improved CLA yield: a 16-fold increase was observed in Polh-pINA1292-sp*opai*-*d12*-7 (7 copies), and a 20-fold increase was obtained by the best-performing transformant, Polh-pINA1292-sp*opai*-*d12*-16 (8 copies), compared to the control strain, Polh-pINA1312-*opai*-8. Polh-pINA1292-sp*opai*-*d12*-16 produced CLA to a level of up to 9.8% (w/w) of total fatty acids, 0.38% (w/w) of DCW, and 45 mg/l after 72 h cultivation in a shaking flask with YPD medium (Table 1, Figure 2).

**Table 1:** **Fatty acid composition and accumulation in cellular lipids of *Y***. ***lipolytica *strains**, **cultivated on YPD medium**

**Fatty acid**	**Transformant**					
	**Polh-1312-o *pai *-8**	**Polh-1312-o *pai *-*d12 *-5**	**Polh-1312-spo *pai *-10**	**Polh-1312-spo *pai *- *d12 *-9**	**Polh-1292-spo *pai *- *d12 *-7**	**Polh-1292-spo *pai *- *d12 *-16**
16:0	8.2 ± 0.4	11.2 ± 0.3	9.5 ± 0.6	11.8 ± 0.9	13.9 ± 0.7	13.3 ± 0.5
16:1	11.3 ± 0.7	6.4 ± 0.8	11.3 ± 1.2	10.5 ± 0.7	12.4 ± 0.8	10.7 ± 1.1
16:2	ND	2.6 ± 0.3	ND	1.0 ± 0.0	0.7 ± 0.1	0.4 ± 0.0
17:1	2.2 ± 0.2	2.3 ± 0.1	2.1 ± 0.1	2.4 ± 0.2	2.5 ± 0.2	2.4 ± 0.3
18:0	0.3 ± 0.0	1.3 ± 0.1	1.2 ± 0.0	1.3 ± 0.2	0.6 ± 0.0	1.1 ± 0.1
18:1	49.7 ± 2.0	13.2 ± 0.5	48.8 ± 1.6	25.9 ± 1.2	21.9 ± 0.8	26.4 ± 1.0
18:2	27.8 ± 1.1	62.2 ± 2.2	26.0 ± 1.3	44.8 ± 1.7	40.3 ± 0.9	35.9 ± 0.5
10 *t*, 12*c*-CLA	0.4 ± 0.1	0.7 ± 0.1	1.1 ± 0.2	2.2 ± 0.4	7.8 ± 0.7	9.8 ± 0.5
TFA (% of DCW)	4.2 ± 0.2	4.0 ± 0.1	4.8 ± 0.4	4.5 ± 0.2	3.7 ± 0.1	3.9 ± 0.3

Values are presented as % (w/w) of the total fatty acid levels in yeast transformants. The means ± SD were obtained from three independent experiments. *TFA*, total fatty acid levels; *ND*, not detected.

Fatty acid analysis (Table 1) showed that in all strains containing recombinant delta12-desaturase, a large proportion of oleic acid (OA) had been converted to LA, which was now the major fatty acid. In addition, delta9,12-16:2 was detected as a new fatty acid in *opai*-*d12* co-expressing strains, suggesting that the delta12-desaturase can also use 16:1 as substrate [11].

### Transformants are stable

The stability of the recombinant *Y*. *lipolytica* strains was tested under nonselective conditions by culturing Polh-pINA1292-sp*opai*-*d12*-16 in YPD for 2 weeks (approximately 160 generations). No change was detected in the copy numbers of *opai*, lipid content and fatty acid compositions of Polh-pINA1292-sp*opai*-*d12*-16 obtained in YPD (data not shown), indicating that the best-performing transformant was stable even after 160 generations.

### Production of CLA by fermentation in YNBD-SO media

As *Y*. *lipolytica* can utilize hydrophobic materials, such as plant oils, as carbon source [12] and soybean oil contains a high proportion of LA (60% of total fatty acids, Table 2, medium at 0 h), the production of *trans*-10, *cis*-12 CLA by the best-performing strain was carried out in a 5 L fermenter with YNBD-SO medium [YNBD medium containing 2% (w/v) soybean oil].

**Table 2:** **Fatty acid composition in cellular and extracellular lipids of Polh**-**1292**-**sp*opai***-***d12***-**16 in fermentations with YNBD**-**SO medium**

**Fatty acid**	**Polh-1292-spo *pai *- *d12 *-16**			
	**Cell - 0 h**	**Cell - 38.5 h**	**Medium - 0 h**	**Medium – 38.5 h**
16:0	12.2 ± 0.6	9.3 ± 0.4	9.9 ± 0.5	11.8 ± 0.6
16:1	7.9 ± 0.8	0.9 ± 0.1	ND	ND
16:2	1.7 ± 0.2	ND	ND	ND
17:1	0.3 ± 0.0	ND	ND	ND
18:0	0.9 ± 0.1	3.6 ± 0.2	3.5 ± 0.1	6.3 ± 0.3
18:1	15.7 ± 1.0	20.2 ± 0.7	19.4 ± 0.9	23.0 ± 1.2
18:2	60.6 ± 2.6	14.6 ± 0.6	57.5 ± 2.2	22.5 ± 0.7
18:3	ND	3.4 ± 0.1	8.0 ± 0.5	2.1 ± 0.3
10 *t*, 12*c*-CLA	0.3 ± 0.0	44.3 ± 2.1	ND	30.4 ± 1.1

Values are presented as % (w/w) of the total fatty acid levels in Polh-1292-sp*opai*-*d12*-16. Other fatty acids were C12:0, C14:0, C20:0, C20:1, C20:2, C22:0 and C24:0 (not shown). The means ± SD were obtained from three independent experiments. *ND*, not detected.

**Figure 3:**
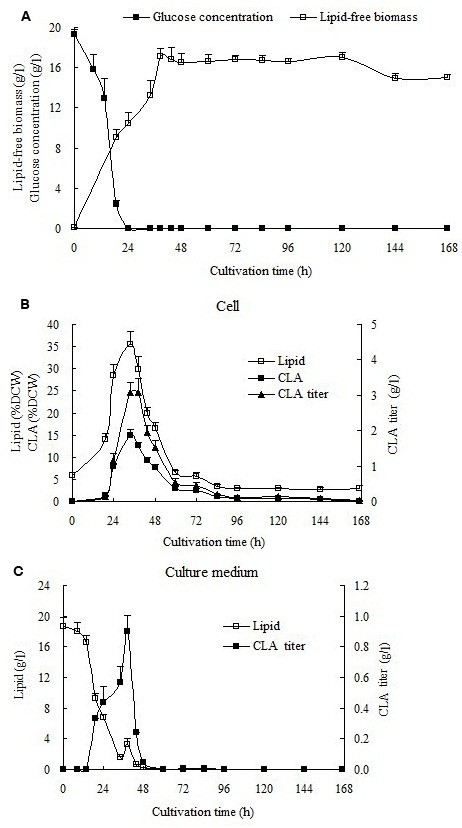
**Growth characteristics, lipid content and CLA production of *Y. lipolytica *Polh-1292-sp*opai-d12-*16 in batch fermentations with YNBD**-**SO medium. **The best-performing strain, Polh-1292-sp*opai*-*d12*-16, was cultivated in YNBD-SO medium for 168 hours under fermentation conditions. (**A**) The growth of *Y*. *lipolytica* was represented by lipid-free biomass calculated after subtraction of the cellular lipids from the total biomass. The glucose concentration was quantified using glucose oxidase in a standard glucose assay kit. (**B**), (**C**) Time course of lipid and CLA contents in cells and culture medium. Lipid and CLA yields in cells are expressed as percentage of total DCW. CLA titre in cells and medium and lipid titres in medium are expressed as g/l. The values represent the mean ± SD of three replicates.

As shown in Figure 3, the cells were in a rapid growth phase from 0 to 38.5 h, with lipid-free biomass concentration increasing from 0.1 g/l to 17.1 g/l (Figure 3A). During that time, glucose was consumed rapidly and depleted by 24 h. Afterwards, the culture was in stationary phase from 38.5 h to 120 h, followed by a decline phase until the end of cultivation (168 h). Both biomass and growth medium were extracted for lipid analysis. Lipid content of the growth medium decreased from 18.7 g/l (the added soybean oil) to zero during the first 60 h of cultivation, indicating complete consumption of the soybean oil (Figure 3C). Meanwhile, total lipid and CLA in cells increased and reached peaks of 35% and 16% (w/w) of DCW, respectively, at 34 h (Figure 3B). The maximum CLA titre in cells was 3.1 g/l both at 34 h and 38.5 h (Figure 3B). Interestingly, CLA was also detected in the growth medium (Figure 3C). During the rapid growth phase, CLA content in growth medium increased continuously and the maximum CLA titre of 0.9 g/l was obtained at 38.5 h. After this time, CLA in cells and growth medium decreased sharply until no more CLA was detected at 168 h and 60 h, respectively.

The fatty acid compositions of cells and culture medium at 0 and 38.5 h of cultivation are shown in Table 2. The LA contents in cells and growth medium decreased from 61% to 15% and from 58% to 23%, respectively. In contrast, the CLA contents in cells and growth medium increased from 0.3% to 44% and zero to 30% respectively, suggesting that LA in the soybean oil added to the medium was efficiently converted to CLA by PAI. In addition, lipid analysis of culture medium at 38.5 h of cultivation revealed that extracellular CLA existed exclusively in free fatty acid form. At the same time point, about 90% of the intracellular CLA existed in free fatty acid form. Total CLA production in the fermenter using YNBD-SO was almost 4 g/l, about 90 times higher compared to that using YPD medium in shaking flask cultures (45 mg/l, Figure 2).

### PAI activity in culture supernatant

To determine whether the CLA detected in culture medium was due to PAI secretion, LA isomerase activity was tested in the growth medium. Yeast cultures were harvested after 24 h by centrifugation. One ml of cell-free medium supplemented with 400 μg free LA (as the substrate for PAI [7]) was incubated at 37°C for 1 h, and then extracted for lipid analysis. The data showed that no LA had been converted to CLA. This suggested PAI secretion had not occurred.

### Cell integrity of *Y*. *lipolytica* in YNBD-SO medium

**Figure 4:**
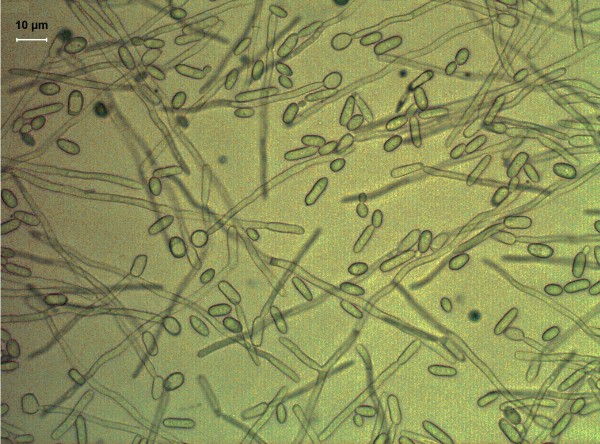
**Cell morphology of *Y. lipolytica *Polh-1292-sp*opai-d12-*16. **Cells were harvested after 24 h of cultivation in YNBD-SO medium. Optical microscopy analysis was performed to determine the integrity of *Y*. *lipolytica* cells under an Olympus System Microscope Model BX51. The images are presented at a 400× magnification.

To determine if the extracellular CLA was due to leakage from the cells, cell integrity of the best-performing strain in YNBD-SO medium was determined using microscopy. As Figure 4 shows, no obvious cell lysis had occurred, and a mixture of yeast-like and short mycelial cells were present at 24 h of fermentation in YNBD-SO.

## Discussion

In our previous study, we constructed a *de novo* CLA biosynthesis system by transforming the oleaginous yeast, *Y*. *lipolytica*, with the recombinant linoleate isomerase gene from *P*. *acnes*[10]. The expression level of *opai* was increased by the optimization of the codon-usage of *pai* and multi-copy integration of the gene. The average copy numbers of o*pai* in pINA1292-*opai* transformants were 12–13 copies/cell, with a maximum of 24 copies/cell, in a strain which was unstable after a few passages of cultivation, and the copy number decreased to 7 copies/cell.

In this study, the average copy number of *opai* integrated in cells using a multi-copy integration vector was estimated at 5–6 copies/cell. The lower number of integrated copies obtained in the present work might result from the much longer integrated fragments (7827 bp) than in previous study (4311 bp). In this study, the best-performing strain, Polh-pINA1292-sp*opai*-*d12*-16, which contained 8 copies of the expression cassette, remained stable in copy numbers and CLA yields (9.8% of total fatty acids). This is consistent with a previous study showing that an average of 10 copies/cell was stable in the Polh-pINA1292 expression system [13]. In addition to multi-copy integration, enhanced function of the promoter can also increase the expression level of *opai*, which appears to be one of the bottlenecks for CLA production in recombinant *Y*. *lipolytica*.

Another potential bottleneck for CLA production in this system is the intracellular concentration of the substrate, LA. Our study showed that co-expression of *d12* with *opai* increased the LA content in cells from 28% to 62% of the total fatty acids; however, the CLA content increased only slightly (from 0.4% to 0.7%) (Table 1). A possible explanation is that the LA being produced by the delta12-desaturase is in an esterified form, which is not a substrate for PAI. An in vitro study has demonstrated that PAI, unlike fatty acyl desaturases and fatty acyl elongases, uses free fatty acid as its only substrate [8]. However, the accumulation of esterified LA may contribute to a slightly increased intermediate pool of free LA and indirectly increase CLA production.

Because PAI is a soluble protein in the cytoplasm, it may compete for the free LA with other cytosolic enzymes that convert free LA to esterified forms of LA (methyl-, CoA-, etc.) in various pathways including oxidation, desaturation, elongation or re-esterification [14]. Competition for the same substrate by the enzymes involved in these pathways may then limit the flux of free LA towards CLA production. Hence, the overall efficiency of the CLA synthesis depends on the expression level of PAI and free LA content in *Y*. *lipolytica*.

In addition, a decrease in LA content (from 62% to 45%) in the o*pai*-*d12* co-expression strain was found when the hp4d promoter located upstream of *opai* was replaced by the hp16d enhanced promoter. The hp4d promoter comports 4 copies of the UAS1B enhancer site upstream of the core promoter, whereas hp16d has 16 UAS1B copies. This decrease in LA is probably due to promoter interference between hp16d driving the *opai* and the weaker hp4d promoter driving the *d12*-desaturase gene.

Due to its ability to hydrolyze extracellular lipids, *Y*. *lipolytica* has been efficiently cultivated on various hydrophobic substrates such as alkenes or lipids to produce many extra- or intra-cellular metabolites of industrial significance [12]. Indeed, in this study, soybean oil, which is rich in LA, was immediately hydrolyzed and absorbed by *Y*. *lipolytica* and free LA was efficiently converted to CLA. This suggests that exogenous LA is a good substrate for PAI, unlike de novo synthesized LA. Additionally, about a quarter of the total CLA production was recovered in the growth medium. As no PAI extracellular activity could be detected in the culture supernatant, these results suggest that CLA present in the medium was not a result of secreted PAI. Furthermore, no cell fragmentation was observed, indicating that CLA was not liberated into the medium by cell lysis. In addition, lipid analysis revealed that the extracellular CLA was in the free fatty acid form.

A possible mechanism that may account for the appearance of CLA in the growth medium is that hydrolysis of the oil, due to the very high activity of lipases in this yeast, may produce free fatty acids faster than they can be incorporated into triacylglycerols and other intracellular lipids. Consequently, as the cell does not accumulate free fatty acids, the latter have to be pumped out of the cells and thus appear in the culture medium as free fatty acids, which include the newly-synthesized CLA. As all the enzymes involved in fatty acid metabolism, including the linoleate isomerase, are able to act on the fatty acids in exogenous soybean oil, CLA production is significantly increased. If *Y*. *lipolytica* could be coaxed into continuously secreting CLA, this transformed yeast strain would be an efficient cell factory able to convert low-cost plant oils into valuable CLA which can be directly collected from the culture medium without biomass extraction processes.

## Conclusion

We constructed a *Y*. *lipolytica* strain able to produce large quantities of *trans*-10, *cis*-12-CLA. This was achieved by combining different genetic engineering approaches to enhance PAI expression and to increase substrate availability.

When the final transformant was cultivated in medium supplemented with soybean oil, 3.1 g CLA/l (16% of DCW) was produced in cells with an additional 0.9 g CLA/l in the growth medium. This result is the highest production of *trans*-10, *cis*-12-CLA to date and shows promise for its large-scale production.

## Methods

### Strains and culture conditions

The auxotrophic strain *Y*. *lipolytica* Polh [15] was used as the PAI expression host, while *Y*. *lipolytica* Polg was used as a control strain for real-time PCR. *Y*. *lipolytica* strains were kindly provided along with the plasmids pINA1312 and pINA1292 [16] by Prof. Catherine Madzak (Institute National de la Recherche Agronomique/AgroParisTech, France). *Escherichia coli* DH5α was used for routine sub cloning and plasmid propagation.

Media and growth conditions for *E*. *coli* were described by Sambrook *et al*. [17], and those for *Y*. *lipolytica* were described by Barth and Gaillardin [18]. YPD and YNBD media were prepared for *Y*. *lipolytica* as described previously [18]. YPD contained 1% (w/v) yeast extract, 2% (w/v) peptone and 2% (w/v) dextrose. YNBD contained 0.17% (w/v) yeast nitrogen base (without amino acids and ammonium sulphate), 0.5% (w/v) ammonium sulphate and 2% (w/v) dextrose. YNBD-SO medium used for fermentation is YNBD with 0.15% yeast extract, 0.5% (w/v) NH_4_Cl, 0.01% (w/v) uracil, 2% (w/v) Casamino acids and 2% (w/v) soybean oil. 2% (w/v) soybean oil in 0.02% (w/v) Tween 80 was prepared as a 10-X sonicated stock emulsion and sterilized by filtration through a 0.22 μm membrane [19].

Typically, cultivation was performed as follows. From YNBD plates, *Y*. *lipolytica* transformants were grown in YNBD (10 ml in 50 ml Erlenmeyer flasks, 200 rpm, 28°C, 48 h). These pre-cultures were inoculated into YPD (50 ml in 250 ml Erlenmeyer flasks) to an OD_600_ of 0.1 and further cultured for another 72 h at 28°C and 200 rpm. Cells were collected by centrifugation (6000 *g*, 5 min) and washed once with de-ionized water, and the pellets were either directly used for real-time PCR and western blot analysis or lyophilized for biomass determination and lipid analysis.

For fermentation experiments, a seed culture was grown in YNBD (150 ml in 500 ml Erlenmeyer flasks, 200 rpm, 28°C, 48 h). The 150 ml seed culture was used to inoculate 3 l YNBD-SO containing 2% (w/v) soybean oil in a fermenter (5 l, BioFlo Celligen 115, New Brunswick). The fermentation was run at 28°C for 7 days. The aeration was at 3 l/min and the stirrer was set at 500 rpm. The pH was maintained at 6.0 with either 3 M KOH or 2 M H_2_SO_4_. Silicone oil was periodically added as an antifoam agent. Ten ml samples were taken at intervals. Cells were harvested by centrifugation (8000 *g*, 15 min) and washed twice with 0.85% (w/v) NaCl and the pellets were lyophilized for biomass determination and lipid analysis. The lipid-free biomass was calculated after subtraction of the cellular lipids from the total biomass. The cell-free culture was used for the LA isomerase activity assay, and heated at 80°C for 10 min (to inactivate lipases) for the glucose concentration assay and lipid analysis.

### Plasmids construction and transformation

Standard protocols were followed for DNA manipulation [17]. Vector pINA1312 and pINA1292 both contain hp4d promoter which comprised four tandem UAS1B copies upstream from a minimal leucine promoter. Previously, UAS1B was found to be an enhancer element and hybrid promoters containing multiple UAS1B sequences successfully amplified the expression levels of *hrGFP* and *lacZ*[20,21]. In this study, the hp16d promoter was constructed by fusing 12 tandem UAS1B enhancer sequences to the hp4d promoter. The four tandem UAS1B sequence fragment, UAS1B_4_ was synthesized (Genscript, Nanjing, China), flanked with *Cla*I-*Hin*dIII-*Spe*I sites on the 5′-end and *Mlu*I-*Xba*I sites on the 3′-end. The synthetic UAS1B_4_ sequence was inserted into pUC57 to form pUC-UAS1B_4_. The UAS1B_4_ fragment was extracted from pUC-UAS1B_4_ by digestion with *Hin*dIII and *Xba*I restriction enzymes and ligated with pUC-UAS1B_4_ cut with *Hin*dIII and *Spe*I, resulting pUC-UAS1B_8_. The construction of pUC-UAS1B_12_ was performed following the same procedures used in the case of pUC-UAS1B_8_. The UAS1B_12_ fragment was extracted from pUC-UAS1B_12_ by digestion with *Cla*I and *Mlu*I restriction enzymes and inserted into pINA1312 and pINA1292 to form pINA1312sp and pINA1292sp, respectively. The codon-optimized LA isomerase sequence (*opai*) [10] was digested with *Pml*I and *Kpn*I and then inserted into pINA1312sp and pINA1292sp to form pINA1312-sp*oPAI* and pINA1292-sp*oPAI*. The delta 12-desaturase sequence (*FADS12*, *d12*) was isolated from *Mortierella alpina* ATCC 32222 cDNA by PCR as previously described [22]. The primers for delta12-desaturase amplification were d12f: (5′-CCGCACGTGATGGCACCTCCCAACA-3′) and d12r: (5′-GGGGTACCTTACTTCTTGAAAAAGACCACG-3′). After amplification, the PCR product was digested with *Pml*I and *Kpn*I and ligated with pINA1312 to form pINA1312-*d12*. The *d12* expression cassette fragment containing the hp4d promoter, flanked with *Eco*RI, was amplified using pINA1312-*d12* as template, with primers d12Expf (5′-GGAATTCTATCGATACGCGTGCATGCTGAG-3′) and d12Expr (5′-CATGGGACACGGGCATCTC-3′). The *d12* expression cassette fragment was digested with *Eco*RI and ligated with pINA1312-*opai*, pINA1312-sp*opai* and pINA1292-sp*opai*, resulting in the expression vectors pINA1312-*opai*-*d12*, pINA1312-sp*opai*-*d12* and pINA1292-sp*opai*-*d12*, respectively. All newly constructed plasmids were screened by restriction enzyme digestion and PCR, and then confirmed by DNA sequencing.

Four new expression cassettes from the plasmids constructed above were transformed into *Y*. *lipolytica* strain Polh, resulting in four recombinant strains, Polh-pINA1312-*opai*-*d12*, Polh-pINA1312-sp*opai*, Polh-pINA1312-sp*opai*-*d12* and Polh-pINA1292-sp*opai*-*d12*. Transformation was performed by the lithium acetate method, as described previously [18]. Transformants were selected by plating on YNBD.

### Real-time PCR and western blotting

Real-time PCR was used to determine the transcript level of *opai* and estimate the copy number of the expression cassettes. The DNA, RNA extraction and real-time PCR were performed as described elsewhere [23,24]. The primer sequences encoding the *opai* were: opaiF (5′-TCCCGATTACGCTGACAAGAC-3′) and opaiR (5′-CTCCATGTCATCCAGCACCAT-3′). Western blot analysis was used to determine the expression level of PAI protein and carried out according to methods described previously [10].

### Lipid extraction and fatty acid analysis

The cells, grown in YPD medium, were collected by centrifugation (6000 *g*, 5 min) and washed once with deionised water, and the pellet was lyophilized prior to lipid extraction. The cells, grown in YNBD-SO medium, were collected by centrifugation (8000 *g*, 15 min), washed twice with 0.85% w/v NaCl-0.5% bovine serum albumin solution and the pellet was lyophilized. The cell-free culture and washing solution were combined for lipid extraction. Lipids from the equivalent weight of freeze-dried cells (20 mg) were directly transmethylated with 10% (v/v) methanolic HCl at 50°C for 3 h [10]. The lipids in medium were extracted twice with a mixture of chloroform/methanol (1:1) and total fatty acids in the medium were similarly methylated with 10% (v/v) methanolic HCl. Free fatty acids in cells and medium were methylated with 20 μL (trimethylsilyl)-diazomethane and 400 μL methanol at 37°C for 30 min. Fatty acid methyl esters (FAMEs) were analyzed by GC [10].

### Stability of the transformants

Strains were grown in YPD for 2 weeks to keep the cells in the exponential phase of growth throughout the experiment. Cells were cultured in 20 ml of medium in 100-ml shaking flasks. Every morning, fresh cultures were inoculated at an initial A_600_ of 0.5 and grown for 8 h. In the evening, fresh cultures were inoculated at an initial A_600_ of 0.2 and grown for 16 h. Samples of the previous culture were used to inoculate the new culture. Under our growth conditions, one cell generation corresponded to 2 h.

### Determination of glucose concentration

Glucose was quantified by using glucose oxidase in a standard glucose assay kit (RSbio, Shanghai. China).

### Extracellular enzymatic activity assay

To determine extracellular LA isomerase activity in YNBD-SO medium, 1 ml cell-free culture, with or without 400 μg free LA, was incubated for 1 h at 37°C. Fatty acids from the reaction system were extracted, methylated and analyzed via GC as described above.

## Competing interests

The authors declare that they have no competing interests.

## Authors' contributions

BXZ and HQC carried out the experiments and drafted the manuscript. ML participated in lipids extraction and data analysis. ZNG, YDS, CR and YQC participated in the experimental design and reviewed the manuscript. HZ and WC conceived the study and reviewed the final manuscript. All authors read and approved the final manuscript.
